# Synthesis and Biological Evaluation of Some Novel 5-[(3-Aralkyl Amido/Imidoalkyl) Phenyl]-1,2,4-Triazolo[3,4-*b*]-1,3,4-Thiadiazines as Antiviral Agents

**DOI:** 10.5402/2012/760517

**Published:** 2012-09-11

**Authors:** Vinod Kumar Pandey, Zehra Tusi, Sumerah Tusi, Madhawanand Joshi

**Affiliations:** ^1^Department of Chemistry, University of Lucknow, Lucknow 226 007, India; ^2^Division of Microbiology, Central Drug Research Institute, Lucknow 226001, India

## Abstract

A series of novel 4-amino-5-mercapto-3-[(3-aralkyl amido/imidoalkyl) phenyl]-1,2,4-triazoles (**5a-d**) were obtained by treating *m*-(aralkyl amido/imidoalkyl) benzoic acid hydrazides (**3a-d**) with carbon disulphide in alcoholic KOH and hydrazine hydrate, respectively. These triazole derivatives were employed in the synthesis of 5-[(3′-aralkyl amido/imidoalkyl) phenyl]-1,2,4-triazolo[3,4-*b*]-1,3,4-thiadiazines (**6a-d**). The newly synthesized compounds were evaluated for their antiviral activity against two animal viruses, namely, *Japanese encephalitis virus* (JEV) strain P20778 and *herpes simplex virus-1* (HSV-1) strain 753166.

## 1. Introduction

 Heterocycles bearing a symmetrical triazole or 1,2,4-triazole scaffold are the structural element of many drugs that have diverse pharmacological activity. The 1,2,4-triazole derivatives are extensively applicable in medicine, namely, alprazolam (tranquilizer), estazolam (hypnotic, sedative, and tranquilizer), rilmazafone (hypnotic, anxiolytic, used in the case of neurotic insomnia), benatradin (diuretic), trapidil (hypotensive), trazodone (antidepressant, anxiolytic), etoperidone (antidepressant), nefazodone (antidepressant, 5-HT2A-antagonist), anastrozole (antineoplastic, nonsteroidal aromatase inhibitor), letrozole (antineoplastic, aromatase inhibitor), ribavirin (antiviral), fluconazole, itraconazole, terconazole (antifungal), and so forth [1]. Furthermore, biheterocyclic compounds bearing a triazole moiety and another heterocyclic moiety combined in one molecular union are also eminent to possess broad spectrum of biological activities, one such imperative being triazolo thiadiazine derivatives [2]. Substituted 1,2,4-triazolo-1,3,4-thiadiazines are reported to possess antifungal, antibacterial, and anticancer activities [3–6]. Additional bioactivity shown by such molecules includes antitubercular, anti-inflammatory, and antimolluscicidal [7, 8]. Significant antiviral activity of such compounds has not been attained however, but the extensive range of the biological effects exhibited by them has engrossed scientists to work on their antiviral effects [9, 10]. Moreover, reports on the activity of triazolo thiadiazines on viruses such as *herpes simplex virus* (HSV-1) and *Japanese encephalitis virus* (JEV) are rare. With no established antiviral therapies available, the only way of prevention is vaccination that is in vogue since the safety of their administration is questionable in certain individuals [11]. Our current endeavor in this direction is therefore the synthesis of a series of 5-[(3′-aralkyl amido/imidoalkyl) phenyl]-1,2,4-triazolo[3,4-*b*]-1,3,4-thiadiazines to assess their antiviral effects which may offer a better perspective in the development of a safer, affordable, and potent vaccine against these two deadly animal viruses. 

## 2. Results and Discussion

A series of 5-[(3′-aralkyl amido/imidoalkyl) phenyl]-1,2,4-triazolo[3,4-*b*]-1,3,4-thiadiazines were synthesized. Although benzoic acid is not a very good nucleophile, moderate yields of *m*-substituted benzoic acids **1** were obtained when benzoic acid was treated with an amido/imido alcohol in concentrated H_2_SO_4_. Reaction of the amidoalkylated benzoic acids with phosphorus pentachloride afforded *m*-(aralkyl amido/imidoalkyl) benzoyl chlorides. Thionyl chloride may alternatively be used for this step. Treatment of acid chlorides with hydrazine hydrate endowed *m*-(aralkyl amido/imidoalkyl) benzoic acid hydrazides. The acid hydrazides were further converted into their corresponding potassium dithiocarbazinates which on cyclization with hydrazine hydrate ensued 4-amino-5-mercapto-3-[(3-aralkyl amido/imidoalkyl) phenyl]-1,2,4-triazoles. Condensation of triazoles with benzoin in presence of polyphosphoric acid yielded the final compounds **6**. These compounds were characterized by their elemental analysis, IR, ^1^HNMR, and mass spectral data incorporated in Table 1. The compounds were also subjected to bioevaluation upon *Japanese encephalitis virus* and *herpes simplex virus-1.* The antiviral activity data has been incorporated in Table 2.

## 3. Conclusions

**Scheme 1:**
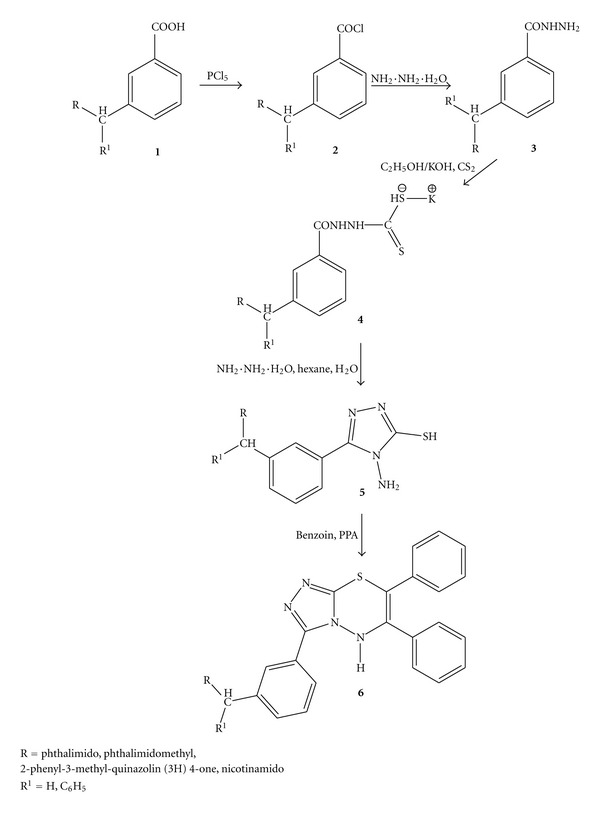


Compound **6c**, having R = H and R^1^ = 2-phenyl-3-methyl-quinazolin (3H) 4-one, displayed moderate anti-JEV activity while the other three compounds, namely, **6a**, **6b**, and **6d** containing phthalimido, phthalimidomethyl, and nicotinamido substituents, respectively, were found insignificantly active at the same dose level. It is quite predictable that a larger substituent like 2-phenyl-3-methyl-quinazolin (3H) 4-one is mainly responsible for exerting anti-JEV activity. On the contrary, three compounds, namely, **6b**, **6c**, and **6d** displayed some activity against HSV-I. The activity percentage ranged from 10 to 23%. From the available biological activity data of the compounds, it can be concluded that unless a large number of such compounds with greater number of variations at two positions are synthesized their potentialities cannot be predicted with great certainty (Scheme 1).

## 4. Pharmacological Activity

### 4.1. Antiviral Activity

Compounds **6** belonging to the triazolo-thiadiazines series were subjected for their assay against two animal viruses, namely,* Japanese encephalitis virus* (JEV) (strain P20778), an RNA virus of high pathogenicity, and *herpes simplex virus-1* (HSV-1) (strain 753166), a DNA virus, originally obtained from National Institute of Virology, Pune (India).

### 4.2. Materials and Methods

#### 4.2.1. Maintenance of *Japanese Encephalitis Virus* (JEV)

 It was properly maintained by intracerebral passages in 1–3 day(s) old suckling albino Swiss mice. The brains of the infected mice with specific paralytic symptoms were triturated and a 10% homogenate (m/V) was made in phosphate buffered saline (PBS) of pH 7.2. The mean lethal dose (LD_50_) of the virus in mice was calculated before each experiment. 

#### 4.2.2. Maintenance of *Herpes Simplex Virus-1* (HSV-1)

It was maintained in 5-6 g albino Swiss mice following the same route as for JEV; a 10% virus homogenate (m/V) was prepared and LD_50_ was calculated as for JEV.

#### 4.2.3. Maintenance of Cells

Vero cells were maintained in minimum essential medium (MEM) (Sigma, USA) with 10% foetal bovine serum (FBS) (Gibco, USA); 100 units of penicillin, 100 *μ*g of streptomycin, and 40 *μ*g of gentamycin were added per mL of the medium. 

#### 4.2.4. Cytotoxicity Test and Antiviral Assay *In Vitro*

Cytotoxicity and antiviral assays of the compounds were performed by the standard method [12]. The experiments were performed in 96-well tissue-culture plates. Equal volumes of maintenance medium and compound solution were poured into each well; concentration of 500 *μ*g mL^−1^ of the compound tested was applied into the first well. Successive dilution by factor 2 was performed in further wells: the compound concentration in the 8th well was 1.9 *μ*g mL^−1^. The treated cultures were incubated for a period of 24 h at 37°C and then observed microscopically for evidence of cytotoxicity, such as distortion, swelling, and sloughing of cells [13–16]. For the antiviral activity, 0.1 mL of the virus (10TC ID_50_  mL^−1^, that is, the dilution previous to 1TC ID_50_, which is the virus dilution that shows 50% cytopathic effect, where TC ID_50_ is 50% tissue-culture infectious dose) was allowed to adsorb onto cell monolayers for 90 min at 37°C [17]. The unadsorbed virus was removed by washing with 0.1 mL of MEM and then 0.1 mL of MEM with 2.5% FBS was filled into each well. Nontoxic concentration of the compound tested, ranging from 3.6 to 125 *μ*g mL^−1^ of the compound, was added into each well. Each dilution was tested in duplicate, keeping separate the virus control and cell control (containing only MEM with 2.5% serum). The culture plates were incubated at 37°C for 72 h and examined microscopically for evidence of cytopathogenicity caused by the virus and its inhibition by the examined compound.

## 5. Experimental Section

Melting points were determined in open capillaries using a Toshniwal melting point apparatus (Japan), and the values recorded are therefore uncorrected. IR spectra in KBr were recorded in the 4000–400 cm^−1^ range using KBr discs on FTR IR 8201 VC Perkin Elmer Spectrophotometer model 337(USA). The NMR spectra were recorded on a Varian 60 D instrument (200 MHz) (USA) using MeOH/DMSO-d_6_. TMS was used as internal standard (*δ* in ppm). Mass spectra of compounds were run on a Hitachi-Elmer RMV-7 spectrometer at 70 eV and FAB mass spectra were recorded on JEOL SX 102/DA-600 Mass Spectrometer/Data System using Argon/Xenon (6 KV, 10 mA) as the FAB gas. Elemental analyses were performed on Carlo-Erba-1108 instrument or Elementar's Vario *EL III* microanalyzer. C, H, and N values were calculated as per the atomic weight of C = 12.01, H = 1.008, N =14.007, O = 15.999, F = Cl = 35.453, and Br = 79.90. The values obtained for each element were expressed as a percentage of the total molecular weight of the compound and agreed with the calculated ones. Purity of the compounds was checked by TLC on silica gel plates. All reagents were purchased from Merck and Ranbaxy. Synthesis of amido alcohols [18] and polyphosphoric acid [8, 19] was accomplished according to the previously reported literature procedures.

### 5.1. *m*-(Aralkyl Amido/Imidoalkyl) Benzoic Acids (**1**, Table 1)

This is an example of C-amido/imidoalkylation reaction [20, 21]. A mixture of benzoic acid and an amido/imido alcohol (0.2 mol) each in minimum quantity of concentrated sulphuric acid (20 mL) was stirred for 3 h and subsequently left overnight under refrigeration. Having poured this mixture into ice-cooled water, (100 mL), the precipitate formed was filtered off, washed repeatedly with water, and treated with 10% aqueous sodium bicarbonate solution. It was stirred till the effervescence ceased, filtered, and the filtrate was neutralized with HCl resulting in the precipitation of the amidoalkylated product. Solid was filtered off, dried over calcium chloride in vacuum, and crystallized from appropriate solvent to obtain the desired product. 

### 5.2. *m*-(Aralkyl Amido/Imidoalkyl) Benzoyl Chlorides, **2**

 A mixture of *m*-(aralkyl amido/imidoalkyl) benzoic acid **1** (0.1 mol) and phosphorus pentachloride (0.15 mol) in anhydrous benzene (50 mL) was refluxed for 2 h under anhydrous condition. Benzene was distilled off under diminished pressure and the acid chloride thus obtained was used for further reaction without any purification [21]. 

### 5.3. *m*-(Aralkyl Amido/Imidoalkyl) Benzoic Acid Hydrazides, (**3**, Table 1)

*m*-(Aralkyl amido/imidoalkyl) benzoyl chloride **2** (0.05 mol) was cooled to 0°C and cold hydrazine hydrate (0.1 mol) was added to it dropwise with slow stirring. On entire addition, the reaction mixture was stirred vigorously for 30 min and poured into water. The solid obtained was filtered off, washed sequentially with water, and dried over calcium chloride in vacuum. The crude product was recrystallized from ethanol which afforded the desired acid hydrazides [21]. 

### 5.4. *m*-(Aralkyl Amido/ImidoAlkyl) Benzoyl-Potassium Dithiocarbazinates, **4**

A mixture of *m*-(aralkyl amido/imidoalkyl) benzoic acid hydrazide **3** (0.03 mol), potassium hydroxide (0.09 mol), and carbon disulphide (0.09 mol) in absolute ethanol (50 mL) was stirred for 24 h at room temperature. The ensuing reaction mixture was diluted with hexane to double of its volume and was used as such for further reaction. 

### 5.5. ****4-Amino-5-Mercapto-3-[(3′-Aralkyl Amido/Imidoalkyl) Phenyl]-1,2,4-Triazoles, (**5**, Table 1)

A mixture of *m*-(aralkyl amido/imidoalkyl) benzoyl-potassium dithiocarbazinate 4 (0.02 mol), hydrazine hydrate (0.08 mol), and water (20 mL) was refluxed for 4 h. Subsequently, the reaction mixture was cooled and filtered off. The clear filtrate was acidified with acetic acid and the resulting precipitate was neutralized. It was washed with water and dried at 100°C. Recrystallization from appropriate solvent afforded analytically pure sample of the desired product. 

### 5.6. ****5-[(3′-Aralkyl Amido/Imidoalkyl) Phenyl]-1,2,4-Triazolo[3,4-b]-1,3,4-Thiadiazines, (**6**, Table 1)

A mixture of 4-amino-5-mercapto-3-[(3′-aralkyl amido/imidoalkyl) phenyl]-1,2,4-triazole **5** (0.01 mol) and benzoin (0.01 mol) in freshly prepared polyphosphoric acid (PPA) (20 mL) was refluxed for 4 h under anhydrous condition. The reaction mixture was cooled to room temperature and poured into ice-cooled water (100 mL). The resultant solid was allowed to settle down. It was filtered off, washed frequently with cold water, and dried over calcium chloride in vacuo. Recrystallization from appropriate solvent afforded crystals of 5-[(3′-aralkyl amido/imidoalkyl) phenyl]-1,2,4-triazolo[3,4-*b*]-1,3,4-thiadiazines. 

## Figures and Tables

**Table 1:** Characterization data of m-(aralkyl amido/imidoalkyl) benzoic acids **1**, *m*-(aralkyl amido/imidoalkyl) benzoic acid hydrazides **3**, 4-amino-5-mercapto-3-[(3^′^-aralkyl amido/imidoalkyl) phenyl]-1,2,4-triazoles **5**, and 5-[(3^′^-aralkyl amido/imidoalkyl) phenyl]-1,2,4-triazolo-[3,4-*b*]1,3,4-thiadiazines **6**.

Compound	R	R^1^	m.p. (^°^C)	Yield (%)	Calculated. (found)			IR (KBr, cm^−1^)	Mass (FAB+) (*m/z*) (M^+^ + 1)	^ 1^H NMR (CDCl_3_, *δ*)
					C	H	N			
**1a**	Phthalimido	H	237–239	60	68.32 (68.30)	3.94 (3.97)	4.98 (4.95)	3540 (OH), 1700 (C=O acid), 1665 (C=O amide)	282	7.26–8.11 (m, ar-H, 8H), 4.21 (s, NCH_2_, 2H), 11.2 (s, OH, 1H)

**1b**	Phthalimido methyl	H	100–102	50	69.15 (69.12)	4.44 (4.46)	4.74 (4.70)	3543 (OH), 1720 (C=O acid), 1665 (C=O amide)	296	7.25–8.21 (m, ar-H, 8H), 4.21 (m, NCH_2_, 2H), 3.91 (m, ar-CH_2_, 2H), 11.4 (s, OH, 1H)

**1c**	2-Phenyl-3-methyl-quinazolin (3H)4-one	H	130–132	55	74.58 (74.54)	4.90 (4.93)	7.56 (7.54)	3530 (OH), 1695 (C=O acid), 1685 (C=O quinazolone moiety), 1665 (C=O amide), 1642 (C=N)	371	7.12–8.25 (m, ar-H, 13H), 4.23 (m, NCH_2_, 2H), 3.94 (m, ar-CH_2_, 2H), 11.2 (s, OH, 1H)

**1d**	Nicotinamido	C_6_H_5_	118-119	65	72.28 (72.25)	4.85 (4.88)	8.43 (8.42)	3540 (OH), 3390 (NH), 1700 (C=O acid), 1656 (C=O amide), 1656 (C=N)	333	7.12–8.17 (m, ar-H, 13H), 8.15 (brs, CONH, 1H), 5.56 (s, CH, 1H), 11.0 (s, OH, 1H)

**3a**	Phthalimido	H	178–180	55	65.08 (65.03)	4.44 (4.48)	14.23 (14.20)	3316 (NH), 1665 (C=O amide)	296	10.43 (s, NH, 1H), 7.20 (brs, NH_2_, 2H), 7.28–8.25 (m, ar-H, 8H), 4.21 (s, NCH_2_, 2H),

**3b**	Phthalimido methyl	H	162–164	50	66.01 (66.00)	4.89 (4.91)	13.58 (13.53)	3210 (NH), 1667 (C=O amide)	310	10.43 (s, NH, 1H), 7.20 (brs, NH_2_, 2H), 7.25–8.19 (m, ar-H, 8H), 4.26 (m, NCH_2_, 2H), 3.93 (m, ar-CH_2_, 2H).

**3c**	2-Phenyl-3-methyl-quinazolin (3H)4-one	H	114–116	55	71.86 (71.83)	5.24 (5.28)	14.57 (14.55)	3286 (NH), 1685 (C=O quinazolone moiety), 1665 (C=O amide), 1642 (C=N)	385	10.52 (s, NH, 1H), 7.25 (brs, NH_2_, 2H), 7.42–8.29 (m, ar-H, 13H), 4.15 (m, NCH_2_, 2H), 3.91 (m, ar-CH_2_, 2H).

**3d**	Nicotinamido	C_6_H_5_	88–90	60	69.35 (69.30)	5.24 (5.27)	16.17 (16.14)	3390 (NH), 3310 (NH amido), 1669 (C=O amide), 1556 (C=N)	346	10.47 (s, NH, 1H), 7.23 (brs, NH_2_, 2H), 8.35 (brs, CONH, 1H), 7.27–8.27 (m, ar-H, 13H), 5.58 (s, CH, 1H).

**5a**	Phthalimido	H	298-299	50	58.11 (58.07)	3.73 (3.77)	19.93 (19.90)	3390 (NH), 2568 (SH), 1660 (C=O amide), 1640 (C=N)	352	11.26 (s, SH, 1H), 7.15 (brs, NH_2_, 2H), 7.24–8.23 (m, ar-H, 8H), 4.21 (s, NCH_2_, 2H).

**5b**	Phthalimido methyl	H	208–210	45	59.16 (59.14)	4.14 (4.16)	19.17 (19.14)	3389 (NH), 2560 (SH), 1668 (C=O amide), 1645 (C=N)	366	11.31 (s, SH, 1H), 7.21 (brs, NH_2_, 2H), 7.27–8.28 (m, ar-H, 8H), 4.24 (m, NCH_2_, 2H), 3.93 (m, ar-CH_2_, 2H).

**5c**	2-Phenyl-3-methyl-quinazolin (3H)4-one	H	146–148	55	65.44 (65.40)	4.58 (4.61)	19.08 (19.03)	3396 (NH), 2565 (SH), 1685 (C=O quinazolone moiety), 1656 (C=O amide), 1642 (C=N)	441	11.43 (s, SH, 1H), 7.27 (brs, NH_2_, 2H), 7.33–8.25 (m, ar-H, 13H), 4.18 (m, NCH_2_, 2H), 3.96 (m, ar-CH_2_, 2H).

**5d**	Nicotinamido	C_6_H_5_	192–194	60	62.67 (62.63)	4.51 (4.55)	20.88 (20.85)	3396 (NH), 3315 (NH amido), 2568 (SH), 1665 (C=O amide), 1642 (C=N)	403	11.41 (s, SH, 1H), 7.22 (brs, NH_2_, 2H), 7.18–8.23 (m, ar-H, 13H), 8.31 (brs, CONH, 1H), 5.54 (s, CH, 1H).

**6a**	Phthalimido	H	284-285	62	70.57 (70.53)	4.01 (4.05)	13.27 (13.24)	3350 (NH), 1663 (C=O amide), 1585 (C=N)	528	10.28 (brs, NH of thiadiazine, 1H), 6.91–8.23 (m, ar-H, 18H), 4.22 (s, NCH_2_, 2H).

**6b**	Phthalimido methyl	H	>300	45	70.96 (70.92)	4.28 (4.30)	12.93 (12.90)	3356 (NH), 1660 (C=O amide), 1595 (C=N)	542	10.26 (brs, NH of thiadiazine, 1H), 7.12–8.21 (m, ar-H, 18H), 4.21 (m, NCH_2_, 2H), 3.92 (m, ar-CH_2_, 2H).

**6c**	2-Phenyl-3-methyl-quinazolin (3H)4-one	H	75-76	50	74.00 (73.97)	4.58 (4.61)	13.63 (13.61)	3360 (NH), 1680 (C=O quinazolone moiety), 1650 (C=O amide), 1585 (C=N)	617	10.24 (brs, NH of thiadiazine, 6.99–8.19 (m, ar-H, 23H), 4.28 (m, NCH_2_, 2H), 3.99 (m, ar-CH_2_, 2H).

**6d**	Nicotinamido	C_6_H_5_	298-299	57	72.64 (72.61)	4.53 (4.57)	14.52 (14.50)	3396 (NH), 1616 (C=O amide), 1556 (C=N)	579	10.29 (brs, NH of thiadiazine, 1H), 7.20–7.88 (m, ar-H, 23H), 8.15 (brs, CONH, 1H), 5.57 (s, CH, 1H).

**Table 2:** Antiviral activity of compounds **6a**–**6d**.

Anti-JEV *in vitro *					Anti-HSV-1 *in vitro *		
Compound. number	CT_50_ (*μ*g/mL)	EC_50_ (*μ*g/mL)	TI	Inhibition (%)	EC_50_ (*μ*g/mL)	TI	Inhibition (%)
**6a**	500	125	04	10	250	02	10
**6b**	500	125	04	10	250	02	10
**6c**	250	7.8	32	50	125	02	23
**6d**	500	125	04	15	250	02	20

CT_50_: 50% cytotoxic concentration; EC_50_: 50% effective concentration; TI: therapeutic index.
